# Aciculatin inhibits lipopolysaccharide-mediated inducible nitric oxide synthase and cyclooxygenase-2 expression via suppressing NF-κB and JNK/p38 MAPK activation pathways

**DOI:** 10.1186/1423-0127-18-28

**Published:** 2011-05-06

**Authors:** I-Ni Hsieh, Anita Shin-Yuan Chang, Che-Ming Teng, Chien-Chih Chen, Chia-Ron Yang

**Affiliations:** 1 School of Pharmacy, College of Medicine, National Taiwan University, Taipei, Taiwan; 2 Institute of Pharmacology, College of Medicine, National Taiwan University, Taipei, Taiwan; 3 Department of Biotechnology, Hungkuang University, Taichung, Taiwan

## Abstract

**Objectives:**

Natural products have played a significant role in drug discovery and development. Inflammatory mediators such as inducible nitric oxide synthase (iNOS) and cyclooxygenase-2 (COX-2) have been suggested to connect with various inflammatory diseases. In this study, we explored the anti-inflammatory potential of aciculatin (8-((2*R*,4*S*,5*S*,6*R*)-tetrahydro-4,5-dihydroxy-6-methyl-2*H*-pyran-2-yl)-5-hydroxy-2-(4-hydroxyphenyl)-7-methoxy-4*H*-chromen-4-one), one of main components of *Chrysopogon aciculatis*, by examining its effects on the expression and activity of iNOS and COX-2 in lipopolysaccharide (LPS)-activated macrophages.

**Methods:**

We used nitrate and prostaglandin E_2 _(PGE_2_) assays to examine inhibitory effect of aciculatin on nitric oxide (NO) and PGE_2 _levels in LPS-activated mouse RAW264.7 macrophages and further investigated the mechanisms of aciculatin suppressed LPS-mediated iNOS/COX-2 expression by western blot, RT-PCR, reporter gene assay and confocal microscope analysis.

**Results:**

Aciculatin remarkably decreased the LPS (1 μg/mL)-induced mRNA and protein expression of iNOS and COX-2 as well as their downstream products, NO and PGE_2 _respectively, in a concentration-dependent manner (1-10 μM). Such inhibition was found, via immunoblot analyses, reporter gene assays, and confocal microscope observations that aciculatin not only acts through significant suppression of LPS-induced NF-κB activation, an effect highly correlated with its inhibitory effect on LPS-induced IκB kinase (IKK) activation, IκB degradation, NF-κB phosphorylation, nuclear translocation and binding of NF-κB to the κB motif of the iNOS and COX-2 promoters, but also suppressed phosphorylation of JNK/p38 mitogen-activated protein kinases (MAPKs).

**Conclusion:**

Our results demonstrated that aciculatin exerts potent anti-inflammatory activity through its dual inhibitory effects on iNOS and COX-2 by regulating NF-κB and JNK/p38 MAPK pathways.

## Introduction

Natural products have proven to be a valuable source for new therapeutic agents. In a search for anti-inflammatory products, aciculatin (8-((2*R*,4*S*,5*S*,6*R*)-tetrahydro-4,5-dihydroxy-6-methyl-2*H*-pyran-2-yl)-5-hydroxy-2-(4-hydroxyphenyl)-7-methoxy-4*H*-chromen-4-one), was selected. Aciculatin, isolated from whole plants of *Chrysopogon aciculatis*, has been used to treat fever and common cold as a traditional Chinese medicine for centuries. Previous study suggested that aciculatin exhibits cytotoxic effect through DNA binding capacity against transformed human KB cell line [1]. However, the molecular details and the anti-inflammatory effect of aciculatin are still unclear.

Through up-regulation of inducible genes, macrophage can secret numbers of inflammatory mediators that contribute to inflammatory responses, including endotoxin-mediated septic shock [2], rheumatoid arthritis [3,4], asthma [5] and other inflammatory vascular disease [6]. Lipopolysaccharide (LPS), a component of the cell wall of gram-negative bacteria, is known to activate a number of cellular signals in macrophages [7]. The two pro-inflammatory enzymes, inducible nitric oxide synthase (iNOS) and cyclooxygenase-2 (COX-2), which can be induced by LPS or cytokines, are found to work in concert in a number of similar pathophysiological activities and inflammatory disease [8,9]. Under basal condition, the products of iNOS and COX-2, including nitric oxide (NO) and prostaglandins (PGs), are involved in modulation of cellular functions and homeostasis. They are highly regulated by biosynthetic pathways that are responsible for pulsed release of nanomolar concentrations of both mediators [10,11]. However, during inflammation, NO and PGs are released simultaneously in large amounts up to micromolar concentration [12]. Previous study has shown that NO directly increases COXs activity and leads to a remarkable 7-fold increase in PGE_2 _formation [13]; further studies suggest that there is a considerable cross talk between NO and PGs biosynthetic pathways [13,14]. Therefore, a compound with the dual inhibitory effect on iNOS and COX-2 expression would hold tremendous potential in advancing the treatment of inflammatory or chronic immune disorders.

Proinflammatory mediators bind to specific receptors cause transcriptional modulation on many genes involved in the further inflammation process [15]. Targeting the intracellular pathways activated between the receptors and gene expression is an attractive concept to develop new anti-inflamatory therapeutic agent, since different proinflammatory mediators can share common intracellular pathways [16]. A binding site for the universal transcription factor NF-κB has been identified in the promoter regions of both the iNOS [17] and COX-2 [18] genes. Inflammatory mediators such as LPS [19], cytokines [20] or mitogen-activated protein kinase (MAPK) members, such as p38 and c-Jun N-terminal kinase (JNK) [21] stimulate the pathways by activating the inhibitor κB (IκB) kinase (IKK) that phosphorylates IκB and leads to its degradation; the free NF-κB could then be translocated to the nucleus and induces the transcriptions of iNOS [22] and COX-2 [23]. This pathway has been known to modulate a wide variety of inflammatory signaling pathways via the up-regulation of iNOS and COX-2. Hence, it has become an attractive therapeutic target for anti-inflammatory drug developments.

The present study examines the inhibitory effect of aciculatin on the expression of iNOS, COX-2 and elucidates the anti-inflammatory mechanisms in LPS-stimulated RAW264.7 macrophages model. Aciculatin was found to decrease LPS-induced iNOS and COX-2 expression, and this effect was correlated with its inhibitory effect on NF-κB activation. These findings together suggest that aciculatin is a potential therapeutically anti-inflammatory agent.

## Materials and methods

### Reagents and materials

**Figure 1:**
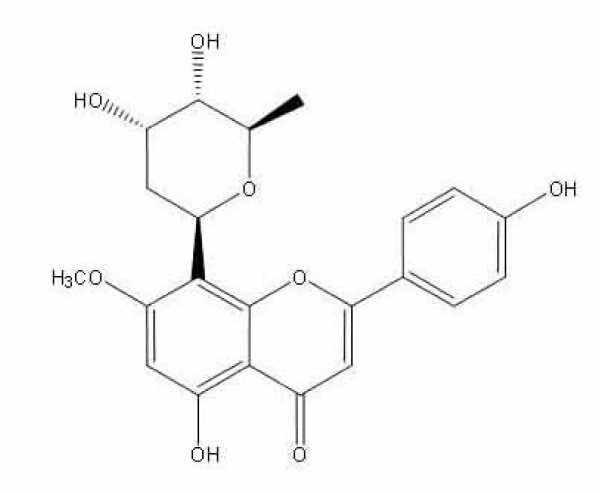
**Chemical structure of aciculatin**.

Aciculatin was extracted and purified by one of our colleagues (Dr. Chien-Chih Chen) to a purity of greater than 98% by HPLC and NMR. Its structure is shown in Figure 1. Mouse monoclonal antibodies against iNOS or GAPDH were purchased from Santa Cruz Biotechnology (Santa Cruz, CA, USA). Rabbit monoclonal antibodies against COX-2, IKKa, and IκBa were purchased from Epitomics Inc. (Burlingame, CA, USA). Rabbit polyclonal antibodies against phosphor-IKKa (Ser180)/IKKb (Ser181), phosphor-ERK1/2 (Thr202/Tyr204), phosphor-p38 (Thr180/Tyr182), phosphor-MKK4 (Ser257/Thr261), MKK4, Phosphor-MKK3/MKK6 (Ser189/207), MKK3, MEK1/2 and rabbit monoclonal antibodies against phosphor-IκBaα (Ser32), phosphor-p65 (Ser536), phosphor-JNK (Thr183/Tyr185), phosphor-MEK1/2 (Ser217/221) were purchased from Cell Signaling Technology (Danvers, MA, USA). Mouse monoclonal anti-NF-κB p65 antibody was obtained from BioVision (Mountain View, CA, USA). Horseradish peroxidase (HRP)-conjugated goat anti-mouse or anti-rabbit IgG antibodies were obtained from Jackson ImmunoResearch Inc. (Cambridgeshire, UK). Prostaglandin E_2 _immunoassay kits were purchased from R&D Systems (Minneapolis, MN, USA). The pGL4.74[hRluc/TK] and pGL4.32[*luc2P/*NF-κB-RE/Hygro] vectors were obtained from Promega Corp. (Madison, WI, USA) and the pEGFP-N1 plasmid was provided by C.-M. Teng (National Taiwan University, Taipei, Taiwan). TurboFect™ *in vitro *transfection reagent was obtained from Fermentas (Burlington, Ontario, Canada). All other chemicals were purchased from Sigma-Aldrich (St. Louis, MO, USA).

### Cell culture

Mouse macrophage cell line RAW264.7 was obtained from the Bioresource Collection and Research Center. Cells were cultured in Dulbecco's modified Eagle's medium (DMEM; Gibco Laboratories Inc.) supplemented with 10% (v/v) fetal bovine serum (FBS; Invitrogen™ Life Technologies, Carlsbad, CA, USA), 100 U/mL of penicillin, and 100 μg/mL of streptomycin (Biological Industries, Kibbutz Beit Haemek, Israel) at 37°C in a humidified atmosphere of 5% CO2 in air. The medium was replaced every 3 days.

### Nitrite and prostaglandin E2 (PGE2) assays

Nitrite production was measured in RAW264.7 macrophage supernatants. Briefly, cells (5 × 10^5 ^cells) were cultured in 24-well plates and stimulated with LPS (1 μg/mL) for 24 h. Then 100 μL of Griess reagent was mixed with 100 μL of the cell supernatant and the optical density at 550 nm was measured. The concentration of nitrite was calculated from a standard curve prepared using known concentrations of sodium nitrite dissolved in DMEM medium. In the prostaglandin E_2 _assay, RAW264.7 macrophages (2 × 10^5^) were cultured in 24-well plates and stimulated with LPS (1 μg/mL) for 24 h, then PGE2 in the culture supernatant was measured using a commercial kit, according to the vendor's instructions.

### Cell viability assay

Cell viability was measured by the colorimetric 3-(4,5-dimethylthiazol-2-yl)-2,5-diphenyl tetrazolium bromide (MTT) assay. Cells (1 × 10^4^) in 100 μL of medium in 96-well plates were incubated with vehicle or test compound for 48 h. Then 25 μL of 1 mg/mL MTT was added and the plate was incubated at 37°C for 2 h. The cells were then pelleted and lysed in 100 μL of dimethyl sulfoxide and the absorbance at 550 nm was measured on a microplate reader.

### Immunoblot analysis

Cells were incubated for 10 min at 4ºC in 20 mM HEPES, pH 7.4, 2 mM EGTA, 50 mM β-glycerophosphate, 0.1% Triton X-100, 10% glycerol, 1 mM DTT, 1 μg/mL of leupeptin, 5 μg/mL of aprotinin, 1 mM phenylmethylsulfonyl fluoride, and 1 mM sodium orthovanadate, then were scraped off, incubated on ice for a further 10 min, and centrifuged at 17,000 g for 30 min at 4ºC. The whole cell extract (60 μg of proteins) was mixed with an equal volume of reducing SDS sample buffer (62.5 mM Tris-HCl, pH 6.8, 2% SDS, 1% glycerol, 300 mM 2-mercaptoethanol, and 0.00125% bromophenol blue) and the mixture was heated at 95ºC for 5 min, electrophoresed on 10% SDS gels, and the proteins were transferred onto polyvinylidene fluoride membranes. Immunoblotting was performed by incubation with the relevant primary antibodies, followed by incubation for 1 h at room temperature with the corresponding HRP-conjugated secondary antibodies, and detection using ECL reagents (Amersham Biosciences) and exposure to photographic film.

### RT-PCR analysis

Total RNA was isolated from cells using TRIzol reagent (Invitrogen). Single-strand cDNA for a PCR template was synthesized from 10 μg of total RNA using random primers and Moloney murine leukemia virus reverse transcriptase (Promega). The oligonucleotide primers used for the amplification were: for mouse iNOS (GenBank Accession No. NM010927), sense (3126-3151), 5'-CCC TTC CGA AGT TTC TGG CAG CAG C-3' and antisense (3598-3623) 5'-GGC TGT CAG AGA GCC TCG TGG CTT TGG-3', with a product of 497 bp, and for mouse COX-2 (GenBank Accession No. NM0111198), sense (149-167) 5'-CAG CAA ATC CTT GCT GTT-3' and antisense (646-666) 5'-TGG GCA AAG AAT GCA AAC ATC-3', with a product of 517 bp. b-actin was used as the internal control; the b-actin primers were sense (613-632), 5'-GAC TAC CTC ATG AAG ATC CT-3' and antisense (1103-1122), 5'-CCA CAT CTG CTG GAA GGT GG-3', with a product of 510 bp. Equal amounts of each reverse-transcription product (1 μg) were PCR-amplified using *Taq *polymerase in 35 cycles of 1 min at 95°C, 1 min at 58°C, and 1 min at 72°C. The amplified cDNA was run on 1% agarose gels and visualized under UV light following staining with SYBR Safe DNA gel stain (Invitrogen).

### Construction of iNOS and COX-2 promoter-luciferase plasmids

The mouse iNOS promoter region from -1588 to +165 bp was amplified from mouse genomic DNA by PCR using the primers 5'-CTCGAGGACTTTGATATGCTGAAATCCATA-3' (sense) and 5'-AAGCTTAGTTGACTAGGCTACTCCGTG-3' (antisense) and ligated into the pGL3-basic vector (Promega, Madison, WI, USA). The mouse COX-2 promoter region from -996 to +70 bp relative to the transcription start was amplified from mouse genomic DNA using the primers 5'-CTCGAGTGGCCAACACAAACACAGTAG-3' (sense) and 5'-AAGCTT CAGTGCTGAGATTCTTCGTGA-3' (antisense). Each 5' amplimer contained a *Xho*I site and each 3' amplimer a *Hin*dIII site, such that the *Xho*I/*Hin*dIII-treated resulting PCR product could be ligated in-frame into the unique *Xho*I/*Hin*dIII site in the pGL3-basic plasmid (Promega). Sequence identities were confirmed using an ABI PRISM 377 DNA Analysis System (Perkin-Elmer Corp., Taipei, Taiwan).

### Transient transfection and reporter gene assay

Cells (1 × 10^6^) in 1 mL of DMEM medium were seeded in each well of 6-well plates one day before transfection. Following the manufacturer's protocol, a mixture of 1 μL of TurboFect™ (Fermentas) and 1 μg of plasmid DNA, pEGFP-N1 plasmid, or pGL4.74[hRluc/TK] vector in 100 μL of DMEM serum-free medium was incubated for 20 min at room temperature, then added to the cells, which were then incubated for 24 h. Transfection efficiency, determined by fluorescence microscopy, was > 60% in all experiments. For the reporter gene assay, 100 μL of reporter lysis buffer (Promega) was added to each well and the cells were scraped off from the dishes. The samples were centrifuged at 16,200 g for 30 s at 4ºC, and the supernatants were collected. Aliquots of cell lysates (20 μL) containing equal amounts of protein (80 μg) were placed in the wells of an opaque black 96-well microtiter plate and 40 μL of luciferase substrate (Promega) was added and the luminescence was immediately measured in a microplate luminometer (Packard, Meriden, CT, USA). To take into account for possible differences in transfection efficiency, the luciferase activity value was normalized using the luminescence from the cotransfected renilla pGL4.74[hRluc/TK] vector (Promega).

### Confocal microscope analysis

Cells were pretreated with aciculatin for 1 h before stimulation with 1 μg/mL LPS for another 1 h. The cells were incubated for 1 h then fixed with 4% paraformaldehyde in PBS for 20 min and permeabilized with 0.5% Trixon X-100 for 15 min. After 1 h incubation with blocking buffer (5% BSA in PBS), cells were incubated with primary antibodies (1:100) in 0.5% BSA for 60 min at room temperature. After 3 × 10 min washes in PBS, the cells were stained for another 60 min with FITC-conjugated secondary antibodies (1:100 dilution in PBS), then were viewed and photographed under a Leica TCS SP5 confocal laser-scanning microscope using appropriate fluorescence filters.

### Data analysis

The data are expressed as the mean ± S.E.M. and were analyzed using one-way ANOVA. When ANOVA showed significant differences between groups, Tukey's post hoc test was used to determine the specific pairs of groups showing statistically significant differences. A *p *value of less than 0.05 was considered statistically significant.

## Results

### Effects of Aciculatin on the LPS-Induced NO and PGE_2 _Production

**Figure 2:**
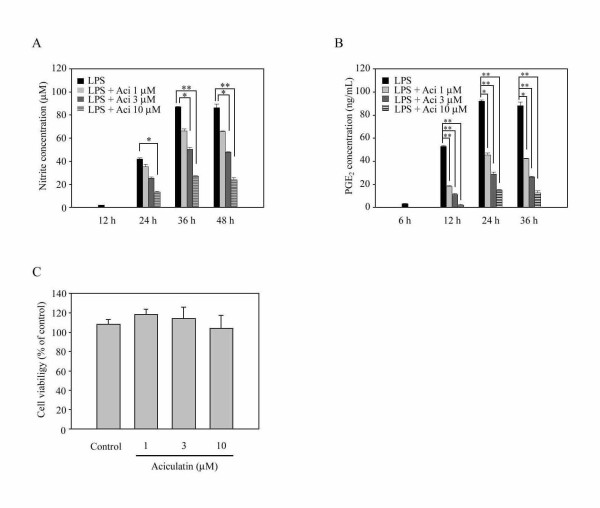
**The concentration-dependently suppressive effects of aciculatin on the LPS-induced production of nitric oxide (NO) and PGE_2_**. RAW264.7 cells (2 × 10^5^) in 24-well plates were incubated with aciculatin (1-10 μM) for 30 min, followed by stimulation with LPS (1 μg/mL) for different periods of time in the continued presence of aciculatin. Then the supernatants were collected and assayed for (A) nitrite and (B) PGE_2_. C. Viability of RAW264.7 cells was determined with treatment of 1-10 μM aciculatin for 48 h in comparison with the control group using the MTT assay. The data are the mean ± S.E.M. for four replicates. * *p *< 0.05 and ** *p *< 0.01 compared to the indicated groups. The experiment was performed four times with similar results.

To investigate whether aciculatin has anti-inflammatory activities, LPS-induced NO and PGE_2 _production was determined in the presence or absence of aciculatin (1-10 μM) in RAW264.7 mouse macrophage cells. Measurement of nitrite as an index of NO production was done by the Griess method. A significant level of nitrite was detected (43.24 ( 0.37 μM) at 24 h after LPS treatment in RAW264.7 macrophages (Figure 2A). The peak level of nitrite concentration (86.72 ( 0.25 μM) was reached after 36 h and remained this level till at least 48 h (85.64 ( 0.82 μM) after LPS treatment. Aciculatin significantly attenuated LPS-induced nitrite production in a concentration-dependent manner (1-10 μM) from 24 to 48 h. Similar inhibitory effect of aciculatin was also found in LPS-induced PGE_2 _production (Figure 2B). Aciculatin concentration-dependently inhibited LPS-mediated PGE_2 _production from 12 to 36 h. This inhibition was not due to cytotoxicity, since none of the treatments had any significant effect on cell viability at 48 h, as assessed using the MTT assay (Figure 2C).

### Aciculatin Inhibits LPS-Induced iNOS and COX-2 Gene and Protein Expression

**Figure 3:**
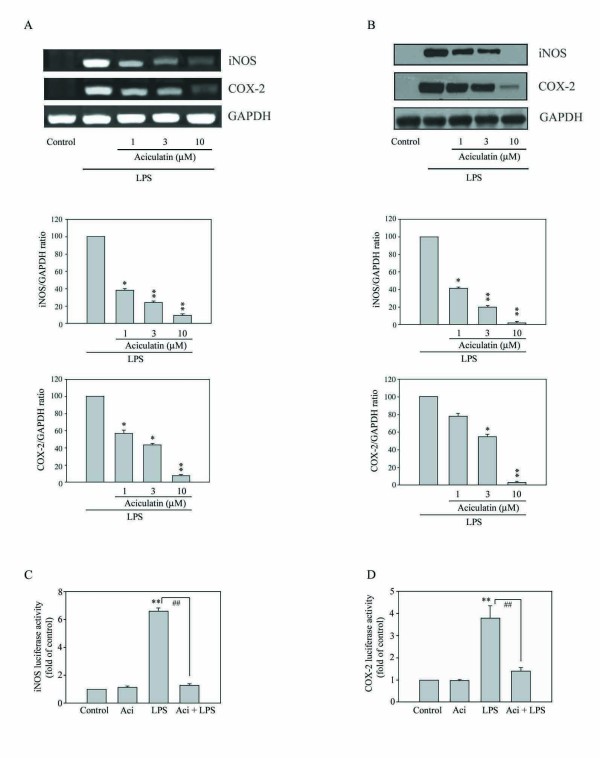
**Aciculatin suppresses the increase of mRNA and protein expression and promoter activity of LPS-induced iNOS and COX-2 in macrophages**. **A. **1 × 10^6 ^RAW264.7 cells were treated with aciculatin (1-10 μM) for 30 min, then stimulated with LPS (1 μg/mL) for 5 h, mRNA of iNOS and COX-2 were measured by RT-PCR. **B. **Treatment of RAW264.7 macrophages with aciculatin (1-10 μM) for 30 min followed by stimulation with LPS (1 μg/mL) for 24 h in the continued presence of aciculatin. Then the cells were harvested and whole cell extracts were prepared for Western blot analysis for the indicated proteins. **C. **Cells (1 × 10^5 ^cells) were transiently transfected with 1 μg of plasmid pGL3-miNOS or pGL3-mCOX-2 for 24 h, then were treated with 10 μM aciculatin for 30 min, followed by stimulation with LPS (1 μg/mL) in the continued presence of the aciculatin for another 24 h. Luciferase activity was then measured as described in the Materials and Methods. The results are expressed as the mean ± S.E.M. for three separate experiments, each with three replicates. * *p *< 0.05 and ** *p *< 0.01 compared with the control group; ## *p *< 0.01 for comparison of indicated groups.

We next to determine whether the inhibitory effect of aciculatin in NO and PGE_2 _production was due to a decrease in expression of iNOS and COX-2. The steady-state levels of iNOS/COX-2 mRNA and proteins following drug treatment were measured by using RT-PCR and immunoblot assays. LPS treatment was shown to induce extensive iNOS and COX-2 mRNA (Figure 3A) and proteins expression (Figure 3B), respectively. Aciculatin markedly decreased LPS-induced iNOS and COX-2 mRNA and protein levels in a concentration-dependent manner in RAW264.7 macrophage cells. To further study the effect of aciculatin on iNOS, COX-2 gene expression, cells were transiently transfected with reporter plasmids containing the promoters for mouse iNOS and COX-2. Treating RAW264.7 macrophages with LPS (1 μg/mL) for 24 h led to an approximately 7.2- or 3.8-fold increase in iNOS (Figure 3C) and COX-2 (Figure 3D) promoter activity, respectively. These effects were significantly inhibited by aciculatin (10 μM) as the levels of iNOS and COX-2 promoter activities returned to basal level. Collectively, these results demonstrate that aciculatin suppressed the expression of iNOS and COX-2 in LPS-stimulated macrophages.

### Aciculatin Suppresses IKK/IκB/NF-κB Signals and NF-κB Nuclear Translocation in LPS-Activated Macrophages

**Figure 4:**
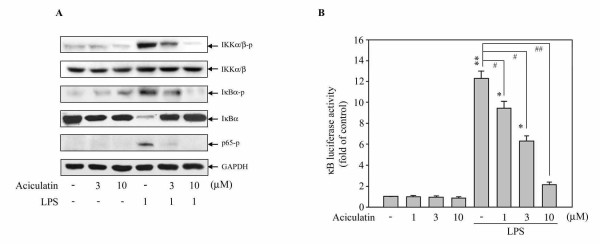
**Aciculatin suppresses NF-κB activation in LPS-activated macrophages**. **A. **RAW264.7 macrophages (1 × 10^6 ^cells) were treated with aciculatin (3, 10 μM) for 30 min and stimulated with LPS (1 μg/mL) for 24 h. Then the cells were harvested and whole cell extracts were prepared for Western blot analysis for the indicated proteins. **B. **Cells (1 × 10^5 ^cells) were transiently transfected with 1 μg of pGL4.32[*luc2P/*NF-κB-RE/Hygro] for 24 h and treated with 1-10 μM aciculatin for 30 min before stimulation with LPS (1 μg/mL) for a further 24 h. Luciferase activity was measured as described in the Materials and Methods. The results are expressed as the mean ± S.E.M. for three separate experiments, each with three replicates. ** *p *< 0.01 compared to the control group; # *p *< 0.05 and ## *p *< 0.01 for comparison of indicated groups.

**Figure 5:**
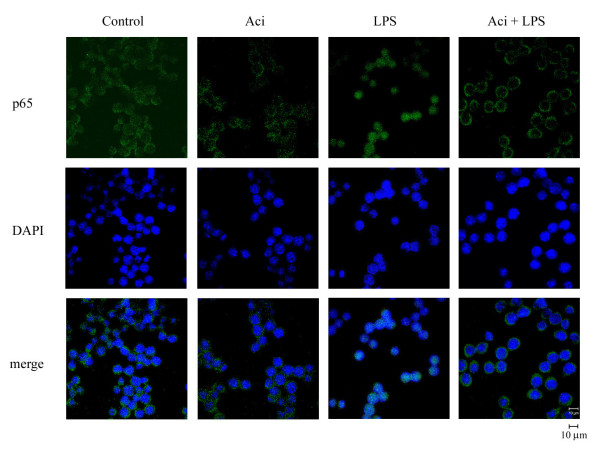
**Effect of aciculatin on LPS-induced NF-κB translocation into the nucleus**. RAW264.7 cells (1 × 10^5 ^cells) were pretreated with aciculatin (10 μM) for 1 h followed by stimulation with LPS (1 μg/mL) for 1 h. Samples were stained by anti-p65 antibody (BioVision) and DAPI, then prepared for confocal microscopy analysis. The results shown are representative of those obtained in four independent experiments. Scale bar = 10 μm.

It has been reported that NF-κB signals regulate the transcription of a wide array of genes, including pro-inflammatory enzymes iNOS and COX-2 in macrophages [18,19]. However, the precise role of aciculatin on regulating NF-κB activation is still unclear. To examine whether aciculatin regulates NF-κB pathways, RAW264.7 macrophages were treated with LPS (1 μg/mL) for 24 h in the presence or absent of aciculatin (3, 10 μM) and levels of the phosphorylated and total forms of IKK(/(, I(B(, and p65 were also examined. LPS treatment not only mediated significant phosphorylation of IKK(/( at serine 180/181, the phosphorylation of IκBa at serine 32, and IκBaα degradation, but also increased the phosphorylation of p65 (Figure 4A). However, 3 μM aciculatin treatment remarkably prevented IKK/IκB/p65 phosphorylation and IκB degradation; 10 μM aciculatin even more significantly rescued to reach basal level. The result of promoter activity assay also showed that aciculatin markedly inhibited LPS-mediated NF-κB promoter activation in a concentration-dependent manner (Figure 4B). Furthermore, the nuclear translocation of NF-κB/p65 was observed under a laser confocal microscope. RAW264.7 macrophages stimulated with LPS showed a dramatic increase in the translocation of NF-κB into the nucleus (Figure 5). In contrast, the LPS-induced NF-κB nuclear translocation was markedly impaired after aciculatin (10 μM) treatment. These results demonstrate that aciculatin significantly inhibited IKK/IκB/NF- κB pathways and NF-κB nuclear translocation.

### Aciculatin Inhibits Phosphorylation of JNK and p38 MAPK in LPS-Stimulated Macrophages

**Figure 6:**
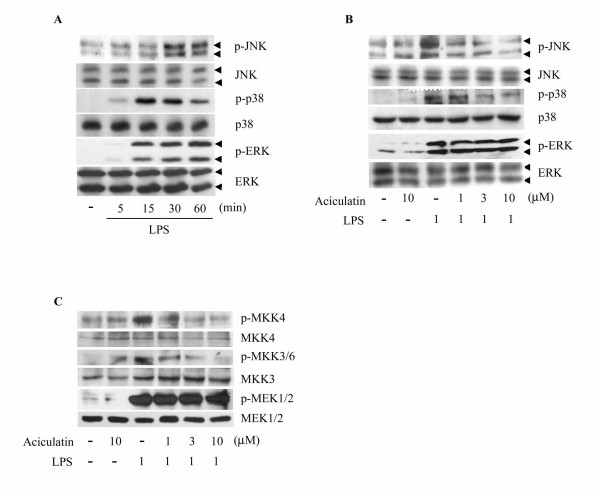
**Aciculatin suppresses JNK, p38 MAPKs and MKK4, MKK3/6 phosphorylation in LPS-activated macrophages**. RAW264.7 cells (1 × 10^6^) were treated with (A) 1 μg/mL LPS for the indicated time periods or (B, C) 1 μg/mL LPS with or without aciculatin (1-10 μM) for 30 min, and cell lysates were then subjected to western blot analysis for the indicated proteins.

MAPKs pathways are also involved in the regulation of proinflammatory mediator expression [21]. Treatment with LPS for 30 min resulted in a significant increase in the phosphorylation of JNK, p38, and ERK compared to the control group (Figure 6A). Aciculatin (1-10 μM) markedly prevented LPS-induced increase of JNK and p38 phosphorylation in a concentration-dependent manner, but not phosphorylation of ERK (Figure 6B). Furthermore, activation of MAPKs (JNK, p38, and ERK) is known to require both tyrosine and threonine phosphorylation by the activated MAPKKs (MKK4, MKK3/6, and MEK1/2), therefore we next to investigate whether aciculatin has inhibitory effect on the activation of MAPKKs. As shown in Figure 6C, LPS treatment mediated a significant increase in the phosphorylation of MKK4, MKK3/6, and MEK1/2. Interestingly, consistent with the inhibitory effect on MAPKs, aciculatin concentration-dependently inhibited LPS-mediated increase of MKK4 and MKK3/6 phosphorylation, but not phosphorylation of MEK1/2.

Together, these results indicate that aciculatin act as a potent anti-inflammatory agent by inhibiting LPS-mediated iNOS and COX-2 synthesis via suppressing NF-κB and JNK/p38 MAPK activation pathways.

## Discussion

In this study, we demonstrated that anti-inflammatory activities of aciculatin, a main component that isolated from *Chrysopogon aciculatis*, in LPS-stimulated RAW264.7 macrophages. Potently dual inhibitory activities against iNOS and COX-2 *in vitro *were shown, suggesting its potential therapeutic usage as a novel topical anti-inflammatory agent.

It has known that LPS elicits strong immune responses, including the production of NO, PGE_2_, and cytokines (e.g. TNF-a, IL-1b, and IL-6) in macrophages [24,25]. Excess amounts of NO and PGE_2 _play a critical role in the aggravation of circulatory shock and chronic inflammatory diseases, such as septic shock [26,27], inflammatory hepatic dysfunction [27], inflammatory pulmonary disease [28], and colitis [29]. Recently, mounting evidence both *in vitro *[13,14] and *in vivo *[30] have indicated an existing cross talk between the release of NO and PGs in the modulation of molecular mechanisms that regulate PGs generating pathway. A group at Monsanto [31] observed that while the production of both nitrite and PGE_2 _was blocked by the NOS inhibitors in mouse macrophages RAW264.7 cells, these inhibitory effects were reversed by co-incubation with the precursor of NO synthesis, L-Arginine. Furthermore, it was also observed that exogenous NO increased COX-2 activity in the IL-1b-stimulated fibroblasts by at least 4-fold, suggested NO directly interacts with COX-2 to cause enzymatic activity. Recent studies indicated that NO *S*-nitrosylates COX-2 in macrophages [9] and cytosolic phospholipase A_2a _(cPLA_2a_) in human epithelial cells [32] and thus activates COX-2 and cPLA_2a_, which provide mechanistic explanation for NO-induced COX-2 activation. In addition, inhibition of iNOS activity by nonselective NOS inhibitors attenuated the release of NO and PG simultaneously in LPS-activated macrophages [33,34], suggested that endogenously released NO from macrophages exerted a stimulatory action on enhancing the PGs production. Conversely, it has been shown that COX activation in turn modulates L-arginine-NO pathway, whereas COX inhibition decreases NOS activity in human platelets [35]. These results are indicative of the cross-talk between NO and PGs pathways. Furthermore, LPS-treated rat gastric mucosa also demonstrated PGE_2 _enhances the release of NO after activation of iNOS [36]; suggest the cross-regulation of PGE_2 _and iNOS existed in LPS-treated condition. Thus, the anti-inflammatory agents that decrease NO and PGs production by simultaneously inhibiting the iNOS and COX-2 gene may have a potentially therapeutic effect in the treatment of inflammatory and infectious diseases. According to our results, aciculatin inhibited LPS-induced NO and PGE_2 _production in a concentration-dependent manner by decreasing the expression of iNOS and COX-2 at both gene and protein level in mouse macrophages. These results suggested that aciculatin might inhibit NO and PGE_2 _production by regulating the transcription molecules of iNOS and COX-2, which could be activated by LPS treatment. In addition, although previous study suggests that aciculatin may have DNA binding activity [37], we noted that reported concentration of aciculatin was higher than we used; suggest that DNA binding effect may not be the major concern in this study. Furthermore, our result of chromatin precipitation assay (supplemental figure 3) clearly demonstrated that aciculatin directly inhibited LPS-induced NF-κB binding to the promoter of COX-2 and iNOS.

Many studies have demonstrated that LPS induces IKK/IκB/NF-κB pathway to stimulate the production of inflammatory cytokines, chemokines, and proinflammatory enzymes (e.g. iNOS and COX-2) [38-40]. The promoter of the iNOS and COX-2 genes are known to contain two transcriptional regions, an enhancer and a basal promoter [41]. There are several binding sites for transcription factors, including NF-κB, which are located in both the enhancer and basal promoter [42]. NF-κB binding site has been identified on the murine iNOS and COX-2 promoters as well and has been observed to play a role in the LPS-mediated induction of iNOS and COX-2 in macrophages [43]. Under unstimulated condition, NF-κB is located in the cytosol and is bound to the inhibitory IκB protein. The activation of NF-κB in response to LPS stimulation leads to increase of nuclear translocation and DNA binding ability, followed by phosphorylation, ubiquitination, and proteosome-mediated degradation of IκB proteins [38-40]. Our results demonstrated that aciculatin has the ability to inhibit the LPS-induced phosphorylation of IKKa/b, IκBa, p65 and IκBa protein degradation as well as p65 nuclear translocation. LPS-mediated iNOS, COX2, and NF-κB promoter activations were also markedly inhibited by aciculatin as shown in the promoter activity assay. These results clearly demonstrated that aciculatin suppresses LPS-induced NF-κB-dependent signals to regulate iNOS and COX-2 expression.

In addition to NF-κB, LPS is a potent activator of MAPK pathways [44]. MAPKs not only play an important role in the LPS-mediated expression of iNOS and COX-2 in mouse macrophages [38-40,44], but also regulate cytokine release [21]. However, using specific inhibitors, different groups [45,46] demonstrated that treatment of MEK1/2 inhibitor, PD98059, was not observed significantly inhibitory effect on NO production and iNOS protein expression in LPS-activated macrophages, suggesting activation of ERK may not the major modulate pathway in LPS-induced NO production [45]. In this study, aciculatin treatment markedly suppressed LPS-stimulated phosphorylation of MAPKKs (MKK4 and MKK3/6) and MAPKs (JNK and p38), these results suggest that suppression of JNK/p38 MAPK phosphorylation by aciculatin might also be involved in inhibition of the LPS-induced production of pro-inflammatory substances in RAW 264.7 cells.

In conclusion, our observations support the evidence that aciculatin exerts anti-inflammatory effect by inhibiting the expression of LPS-stimulated iNOS and COX-2 inflammation-associated genes via suppression of transcription factor NF-κB activation and JNK/p38 MAPKs pathway. In view of the fact that NO and PGE_2 _play important roles in mediating inflammatory responses, it suggests that aciculatin might be a potential anti-inflammatory agent.

## Competing interests

The authors declare that they have no competing interests.

## Authors' contributions

INH carried out the main experiment. ASYC performed partial western blot assays. CMT contributed to the scientific discussion. CCC provided the purified aciculatin compound. CRY designed experiments and finalized the manuscript. All authors read and approved the final version of the manuscript.
